# Idiopathic Spinal Cord Infarction: A Case Report of an Unusual Diagnostic Dilemma and Therapeutic Challenge

**DOI:** 10.7759/cureus.95578

**Published:** 2025-10-28

**Authors:** Safae Dehbi, Manal Arfaoui, Bouchra Armel, Hamza El Hamzaoui, Mustapha Alilou

**Affiliations:** 1 Emergency Department, Ibn Sina University Hospital, Mohammed V University, Rabat, MAR

**Keywords:** anterior spinal artery, case report, idiopathic, rehabilitation, spinal cord infarction

## Abstract

We report a 23-year-old male patient with acute respiratory distress and progressive quadriparesis following a football match, without preceding trauma. Initial imaging and cerebrospinal fluid studies were unremarkable. Spinal MRI revealed an infarction in the anterior spinal artery territory (C2-D1). Extensive etiological workup was negative. Despite treatment with corticosteroids and intravenous immunoglobulin, neurological recovery was limited. The patient required tracheostomy and intensive rehabilitation, eventually regaining partial motor function.

Idiopathic spinal cord infarction (SCI) is rare, particularly in young patients. Diagnosis is challenging due to overlap with other causes of acute myelopathy. No evidence-based treatments exist; management relies on supportive therapy and early rehabilitation. Clinicians should maintain a high index of suspicion for SCI even in young patients. Early diagnosis and multidisciplinary rehabilitation remain crucial for achieving optimal outcomes.

## Introduction

Spinal cord infarction (SCI) is a rare condition caused by a sudden loss of blood flow to the spinal cord, leading to tissue damage and neuronal death. Although the spinal cord is richly vascularized, it has relatively poor collateral circulation, making it vulnerable to ischemic injury. SCI represents about 1.2% of all strokes and 6% of acute myelopathies [1]. The true incidence is difficult to establish, but population-based studies estimate that it ranges between 1.5 and eight cases per 100,000 person-years, depending on the population studied and the diagnostic methods used [2,3]. SCI can occur in various clinical settings, especially around the time of medical procedures, and is often linked to aortic disease, aortic surgery, diabetes, hypertension, or hyperlipidemia [1]. Spontaneous, non-traumatic SCI is rare in young people, with an incidence as high as eight per 100,000 [2]. The clinical presentation is highly variable, ranging from mild sensory disturbances to complete quadriplegia, often mimicking alternative diagnoses such as transverse myelitis or demyelinating disorders [4]. Because of this overlap, SCI is frequently underdiagnosed or misdiagnosed, leading to delays in treatment and worse functional outcomes [4]. Early recognition and supportive management, including rehabilitation, are therefore critical for improving prognosis [5,6].

## Case presentation

We report the case of a 23-year-old male patient with no significant past medical history who was admitted to the Ibn Sina Emergency Department for respiratory distress. Ten hours before admission, following a football match (without direct trauma), the patient noted distal paresthesias in all four limbs, followed by progressive weakness. There was no history of fever, recent infection, or gastrointestinal symptoms.

On admission, he was tachypneic (40 breaths/min) and hypoxemic (oxygen saturation (SpO₂), 70%) but showed no signs of increased work of breathing. He was conscious (Glasgow Coma Score (GCS) 15), hemodynamically stable (heart rate 90 bpm, blood pressure 130/80 mmHg, with no signs of hypoperfusion). Motor strength was reduced: 4/5 in the right lower limb and 3/5 in the left lower limb, with distal upper-limb weakness progressing proximally. Superficial sensation was decreased in a cape-like distribution affecting both upper limbs, with impaired proprioception and vibration sense below C5. The patient reported subjective paresthesias in both hands and feet. Deep tendon reflexes were globally absent, and plantar responses were mute bilaterally. Muscle tone was decreased (flaccid paraparesis). Despite oxygen supplementation in a semi-sitting position, hypoxemia persisted. Ninety minutes after admission, he experienced neurological deterioration: GCS dropped to 9 (E2V2M5), lower limb power declined further (right 2/5, left 1/5), and he developed hypoventilation. Arterial blood gas showed severe respiratory acidosis (pH 7.10, partial pressure of carbon dioxide (pCO₂) 85 mmHg, partial pressure of oxygen (pO₂) 75 mmHg, bicarbonate (HCO₃⁻) 26 mmol/L, base excess −1.5). He was intubated using rapid-sequence induction (etomidate 20 mg, rocuronium 80 mg) and placed on mechanical ventilation.

**Figure 1:**
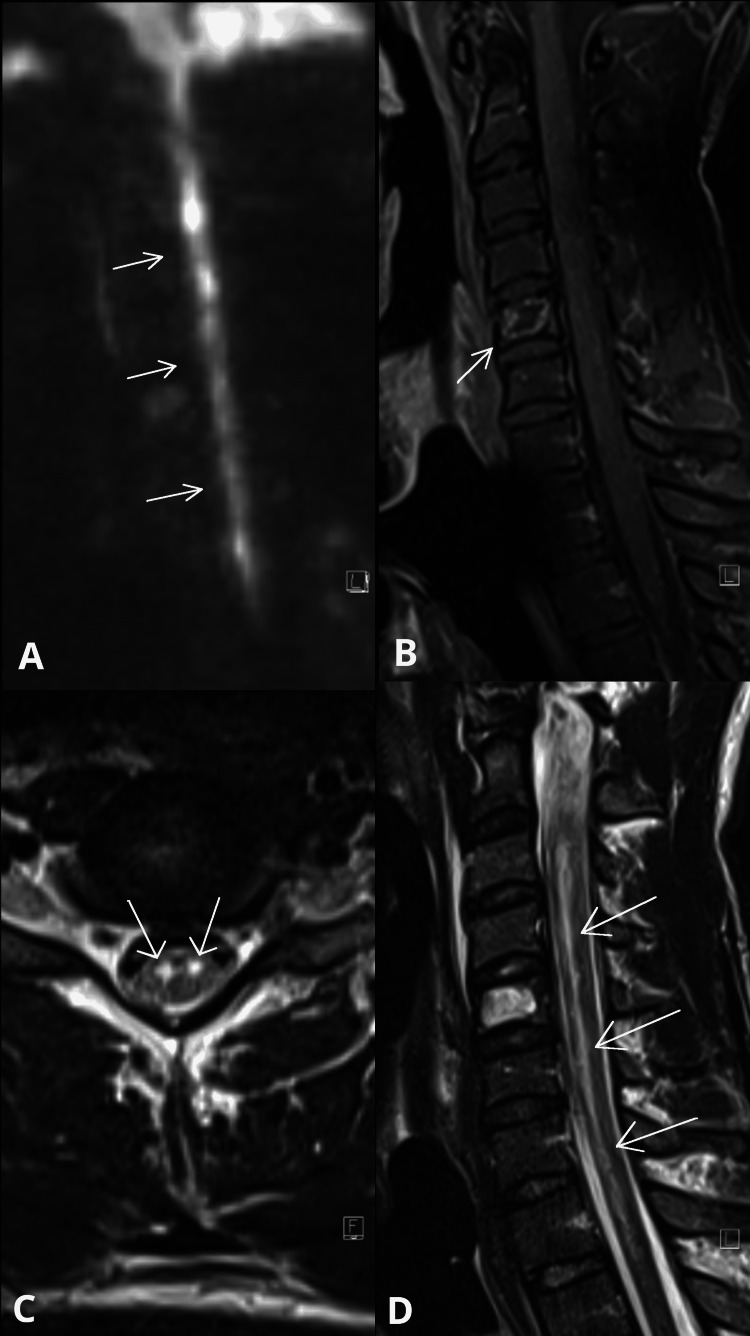
MRI of the cervical spine demonstrating intramedullary spinal cord lesions (A): Sagittal diffusion-weighted imaging (DWI) sequence showing a hyperintense linear lesion extending from C2 to D1, consistent with acute infarction in the anterior spinal artery territory. (B): Sagittal gadolinium-enhanced T1 image demonstrating C5 vertebral body lesion with peripheral enhancement after gadolinium administration. (C): Axial T2 section showing central hyperintensity involving the anterior two-thirds of the cord, with relative sparing of the posterior columns, consistent with anterior spinal artery infarction. (D): Sagittal T2-weighted image highlighting the involvement of the mid-to-lower cervical cord with well-demarcated intramedullary hyperintensity.

Emergency CT brain and spine, and CT angiography of the carotid and vertebral arteries were normal. The following day, MRI (sagittal T1, axial T2-weighted, diffusion-weighted imaging (DWI), susceptibility-weighted imaging (SWI), 3D fluid-attenuated inversion recovery (FLAIR), and 3D time-of-flight (TOF)) revealed a longitudinal hyperintense lesion from C2-D1 in the anterior spinal cord territory, showing restricted diffusion and focal ring enhancement at C5, consistent with a subacute anterior spinal artery infarction (see Figure 1). Notably, 3D TOF excluded carotid or vertebral arterial dissection. A multidisciplinary team (neurology, neuroradiology, neurosurgery) reviewed the images and agreed they were not compatible with Spinal Cord Injury without Radiographic Abnormalities (SCIWORA), which typically shows features of traumatic contusion without vascular territory distribution.

Cerebrospinal fluid (CSF) analysis following lumbar puncture was clear, with no albuminocytologic dissociation: white blood cells 3/mm³, red blood cells 3/mm³, protein 0.29 g/L, CSF glucose 0.91 g/L (concomitant blood glucose 1.1 g/L). A multiplex polymerase chain reaction (PCR) panel was also negative. Laboratory investigations, including complete blood count, serum biochemistry, coagulation studies, and urinalysis, revealed no abnormalities. A thorough workup was then performed to determine the etiology of the spinal cord infarction. Complement levels (C3 and C4) were normal. Autoimmune and vasculitis markers, including antineutrophil cytoplasmic antibody (ANCA) (C- and P-ANCA), anti-nuclear antibodies, anti-cardiolipin antibodies, and anti-β2 glycoprotein I antibodies, were negative. Infectious workup, including Lyme serology, HIV, and syphilis serology, was negative. Inflammatory markers showed a mildly elevated C-reactive protein (CRP, 54 mg/L) and an erythrocyte sedimentation rate (ESR) of 13 mm/h. Serum protein electrophoresis revealed an inflammatory pattern, accompanied by a slight reduction in the β1-globulin fraction. Procalcitonin was negative, and the homocysteine level was within normal limits (10.74 µmol/L). The patient's lab test results are presented in Table 1.

**Table 1:** Laboratory findings of the patient

Parameter	Result	Normal range
White blood cells (CSF)	3/mm³	0-5/mm³
Red blood cells (CSF)	3/mm³	0/mm³
Protein (CSF)	0.29 g/L	0.15-0.45 g/L
Glucose (CSF)	0.91 g/L	0.6-0.8 of serum glucose g/L
Serum glucose	1.1 g/L	0.7-1.1 g/L
CRP	54 mg/L	<5 mg/L
ESR	13 mm/h	0-15 mm/h
Complement C3	105 mg/dL	90-180 mg/dL
Complement C4	28 mg/dL	10-40 mg/dL
Homocysteine	10.74 µmol/L	5-15 µmol/L
Procalcitonin	0.01 ng/mL	<0.05 ng/mL

Despite these findings indicating the absence of systemic autoimmune disease, infection, or a hypercoagulable state, the patient received a bolus of methylprednisolone at 15 mg/kg for three days without improvement. He was then treated with intravenous immunoglobulin at a total dose of 2 g/kg, divided over four days, as a trial therapy, but again without clinical improvement. These results supported the diagnosis of idiopathic or isolated anterior spinal artery infarction in this young patient.

During his ICU stay, the patient was started on prophylactic aspirin therapy. He required a tracheotomy on day 10 after three unsuccessful extubation attempts. He developed ventilator-associated pneumonia, with microbiological tests confirming *Acinetobacter baumannii*, and was treated with colistin and imipenem for seven days. After several days of motor physiotherapy and rehabilitation, he regained partial muscle strength, with ratings of 3/5 in the right lower limb, 2/5 in the left lower limb, and 2/5 in the upper limbs and distal extremities. He underwent a prolonged ventilator weaning process but kept the tracheotomy cannula.

The patient was discharged from the ICU on day 30 and transferred to a rehabilitation center at the Ayachi Hospital of the Ibn Sina University Hospital, where he continued intensive rehabilitation. At the four-month follow-up, he was able to walk with the assistance of a cane. Muscle strength in the upper limbs had fully recovered (5/5), while the left lower limb remained weak (3/5), with partial recovery of the right lower limb. His condition was graded on the American Spinal Injury Association Impairment Scale as “D” [7].

## Discussion

While spontaneous SCI is a rare clinical condition, its consequences can be devastating and potentially life-threatening. In a population-based study from Minnesota, Qureshi et al. reported an overall incidence of acute SCI of 3.1 per 100,000 person-years (95% confidence interval (CI), 1.6-7.2), with the incidence of spontaneous cases estimated at 1.5 per 100,000 person-years (95% CI, 0.6-3.6) [3]. Several risk factors have been identified in association with SCI, including hypertension, dyslipidemia, diabetes mellitus, smoking, prior stroke or peripheral vascular disease, and obesity [1].

Spinal cord infarction can result from a broad spectrum of causes, which are generally classified as either spontaneous or periprocedural. Among spontaneous cases, vascular obstructions such as atherosclerosis, cardioembolism, aortic dissection or aneurysm, and fibrocartilaginous embolism are most frequently implicated [8]. Trauma, including cervical manipulation or sudden motion, may also precipitate arterial injury leading to infarction [1]. Less common associations include sickle cell disease, arteriovenous malformations, autoimmune vasculitis, and even infections (e.g., HIV, varicella-zoster virus, and Lyme disease), which may act through mechanisms such as viral vasculitis, post-infectious immune activation, or infection-induced hypercoagulability [8]. However, despite recent advances, the idiopathic form of spontaneous SCI remains relatively common, accounting for approximately 68% of spontaneous cases in one large cohort, which presents a significant dilemma in selecting appropriate treatment options [4].

The diagnosis of spontaneous SCI is particularly challenging, and recent studies suggest a high rate of misdiagnosis, with 14% to 16% of patients initially referred for evaluation of transverse myelitis ultimately found to have SCI [4]. Zalewski et al. emphasized these diagnostic difficulties, especially in patients without obvious triggers such as surgery or trauma, and proposed structured diagnostic criteria based on a large cohort of 133 patients evaluated at the Mayo Clinic between 1997 and 2017 [4]. These criteria were derived from detailed clinical, radiological, and laboratory analyses, highlighting acute progression of deficits, characteristic MRI findings, noninflammatory cerebrospinal fluid (CSF), and exclusion of alternative etiologies. Validation showed good specificity, as almost no patients with other myelopathies fulfilled the criteria for definite or probable SCI. Applying the diagnostic framework of Zalewski et al., our case fulfills criterion 1 (rapid evolution to a severe deficit within 12 hours from initial symptom onset), criterion 2A (no extrinsic compression on emergent spine imaging), criterion 2B (supportive MRI pattern with a longitudinal anterior-predominant T2-hyperintense lesion from C2-D1), criterion 2C (specific/confirmatory features with diffusion restriction and focal ring enhancement at C5 consistent with subacute infarction; carotid/vertebral arterial dissection excluded on 3D TOF), criterion 3 (non-inflammatory CSF), and criterion 4 (no more likely alternative diagnosis after comprehensive work-up). Collectively, these findings categorize the presentation as Definite spontaneous spinal cord infarction (imaging-confirmed) according to Zalewski et al. [4].

There are currently no standardized or evidence-based specific treatments for idiopathic spontaneous SCI, largely due to its rarity and diagnostic challenges. Supportive treatments, such as antiplatelet therapy, remain the mainstay of management. When an etiology is identified (such as post-aortic surgery ischemia or vertebral artery dissection), targeted interventions like CSF drainage, endovascular procedures, or antithrombotic therapy may be appropriate. In idiopathic cases, it is more challenging to adopt a single, protocol-driven treatment; however, hemodynamic optimization and risk factor management, such as blood pressure control and statin therapy, remain essential, modeled after acute ischemic stroke care [5]. Recent studies have reported corticosteroid use in 41% of cases to reduce oxidative stress, but it lacks supporting evidence and is associated with significant adverse effects [1]. Similarly, only a small number of patients received intravenous thrombolysis, limiting the ability to evaluate its effectiveness [1]. Rehabilitation should begin as early as possible to maximize neurological recovery. Unlike non-idiopathic cases, early rehabilitation has proven to be more effective in idiopathic SCI [1]. Evidence further indicates that patients who undergo rehabilitation at specialized facilities achieve significantly better outcomes, including lower mortality rates and greater functional independence, compared to those treated in less intensive settings [1]. Moreover, incorporating multidisciplinary approaches, combining physiotherapy, cognitive and psychological support, and advanced interventions, has been shown to produce substantial improvements in motor function, cognitive performance, and overall quality of life [6].

## Conclusions

Idiopathic spontaneous SCI is a rare and diagnostically complex condition, especially in young patients without risk factors. Early detection is essential, even though there are no standardized clinical signs; clinicians should always consider this diagnosis to prevent delays and initiate appropriate treatment promptly. Since evidence-based options are limited, management mainly involves supportive care, with rehabilitation being the key to recovery. Early, intensive, and multidisciplinary approaches provide the best opportunity for better neurological and functional outcomes.
